# Phytohormone and Chromatin Crosstalk: The Missing Link For Developmental Plasticity?

**DOI:** 10.3389/fpls.2019.00395

**Published:** 2019-04-05

**Authors:** Stéphane Maury, Mamadou Dia Sow, Anne-Laure Le Gac, Julien Genitoni, Clément Lafon-Placette, Iva Mozgova

**Affiliations:** ^1^LBLGC, INRA, Université d'Orléans, EA1207 USC 1328, Orléans, France; ^2^BIOSS Centre for Biological Signaling Studies, Institute for Biology III, University of Freiburg, Freiburg, Germany; ^3^ESE, Ecology and Ecosystem Health, Agrocampus Ouest, INRA, Rennes, France; ^4^Department of Botany, Charles University, Prague, Czechia; ^5^Centre Algatech, Institute of Microbiology of the Czech Academy of Sciences, Trebon, Czechia; ^6^Faculty of Science, University of South Bohemia in Ceske Budejovice, Ceske Budejovice, Czechia

**Keywords:** DNA methylation, epigenetics, meristem, robustness, signaling

Plants grow continuously, forming new meristem-derived organs and tissues throughout their post-embryonic life. As sessile organisms, plants need to constantly integrate and reflect environmental fluctuations in their growth and development, which can translate into high level of developmental plasticity in response to environmental changes (Gaillochet and Lohmann, 2015). Alternatively, variable environments can select for robustness, where organisms function across a wide range of conditions with little change in phenotype. Plant growth is then governed by complex interplay of phytohormone signaling, chromatin structure remodeling and gene expression reprogramming. How these regulatory levels are interconnected remains largely enigmatic, but mechanistic evidence of crosstalk between phytohormone signaling and chromatin organization is emerging.

Here we review (1) evidences of molecular mechanisms that mediate the crosstalk between phytohormone signaling, chromatin structure and gene expression (2) how this crosstalk may link to plant developmental plasticity and robustness and finally (3) why meristems may represent central places for this crosstalk allowing plasticity and environmental memory.

## Crosstalk Mechanisms: A Chicken-and-Egg Situation

**Figure 1:**
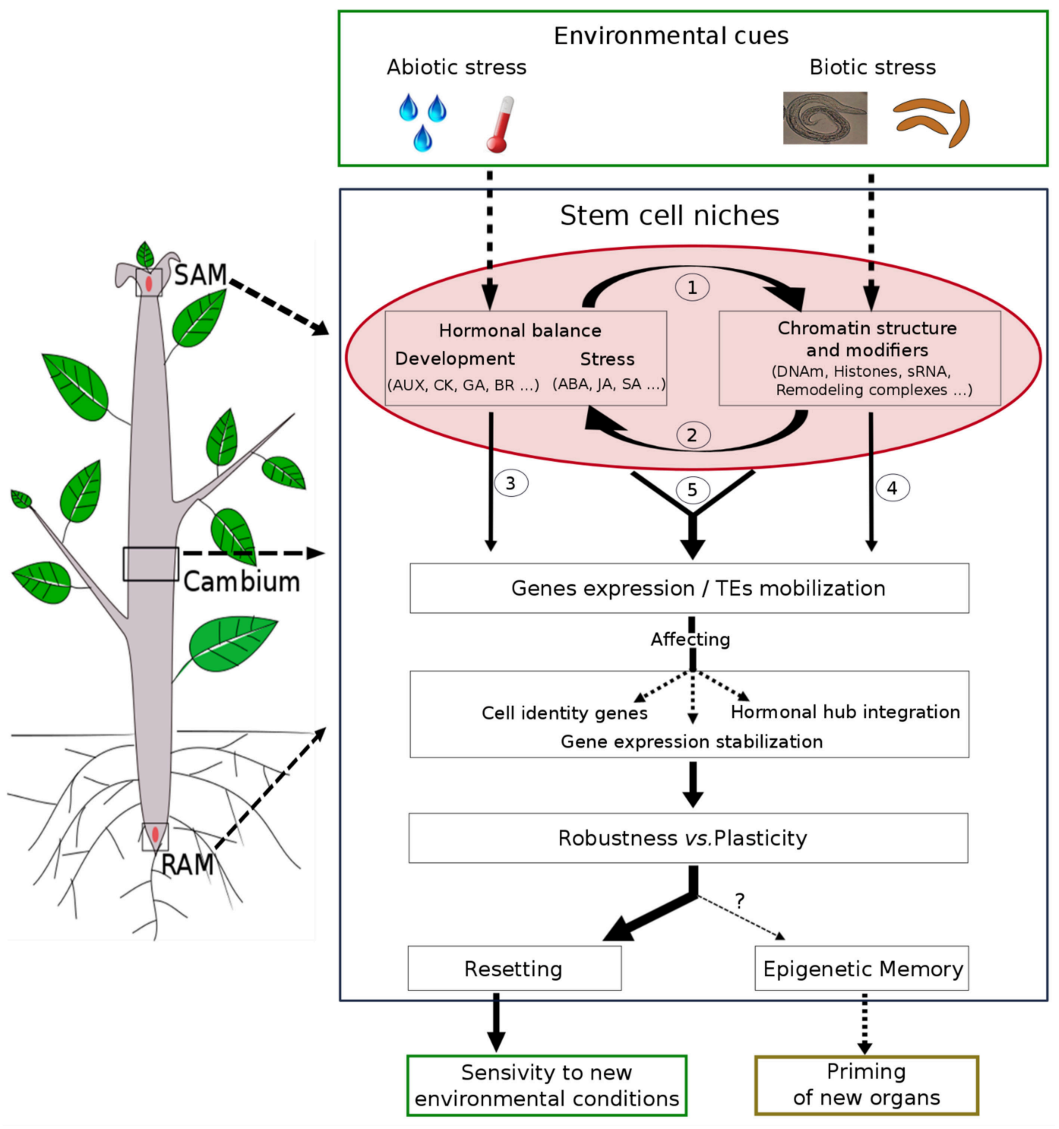
Schematic model of phytohormones and chromatin crosstalk during plant developmental plasticity and robustness. Stem cell niches in SAM, RAM, or cambium are center of morphogenesis giving rise to the aerial and root systems or wood formation in perennials and plasticity in response to various environmental cues. Environmental signals are perceived directly or indirectly by meristems and could affect hormonal balance and/or chromatin structure in a complex crosstalk: (1) hormones can alter chromatin structure and modifiers or (2) chromatin can regulate hormones signaling/biosynthesis. These two mechanisms could then interact separately (3 and 4), jointly or successively (5) affecting genes expression and /or TEs mobilization. Thus, the hormone/chromatin crosstalk can participate in developmental choice (Robustness vs. Plasticity) by controlling cell gene identity in meristems, hormone balance integration, or chromatin stabilization of gene expression. While most of these changes are transient (resetting of hormonal and chromatin modifications) allowing the plant to be respond to new environmental conditions, chromatin states could be maintained through cell division allowing an epigenetic memory and a potential priming of new meristem-derived-organs.

Phytohormone and epigenetic regulation can interact on multiple levels (Figure 1): (1) phytohormone signaling directly affects expression or activity of key chromatin modifiers, (2) chromatin machinery target genes of the phytohormone metabolic/signaling pathways, (3) both players interact on genes involved in developmental or stress responses.

Several examples show that components of phytohormone signaling pathways directly control the activity of key chromatin modifiers such as POLYCOMB REPRESSIVE COMPLEX (PRC) 1 and 2 with histone-methyltransferase activity playing a major role in transcriptional regulation during development (Bratzel et al., 2010; Chen et al., 2010, 2016; Ikeuchi et al., 2015; Mozgová et al., 2017). For example, the brassinosteroid (BR) signaling TFs BRASSINAZOLE-RESISTANT 1 (BZR1) recruits the H3K27me3-demethylase EARLY FLOWERING (ELF) 6 to antagonize the H3K27me3-activity of PRC2, a chromatin modifier, at the flowering repressor *FLOWERING LOCUS C* (*FLC*), preventing precocious floral transition (Yu et al., 2008; Li et al., 2018). Additionally, chromatin complexes can be post-translationally modified by components of phytohormone signaling pathways that influence their activity. For example, abscisic acid (ABA) signaling induces SnRK-mediated phosphorylation of the chromatin remodeling ATPase BRAHMA (BRM), inhibiting its repressive activity at ABA-responsive genes (Peirats-Llobet et al., 2016). These examples demonstrate that activity of chromatin modifiers can be directed to specific loci or directly modulated by phytohormone signaling cascades.

Another possibility is that changes in chromatin structure control phytohormone biosynthesis, signaling and response. Variation in DNA methylation in response to water availability in poplar or among Arabidopsis epigenetic recombinant inbred lines (epiRILs) is associated with changes in jasmonic (JA), salicylic acid (SA) and ethylene responses (Latzel et al., 2012; Lafon-Placette et al., 2018). Similarly, rice plants with reduced H3K27me3 exhibit significant differences in the auxin indole-3-acetic acid (IAA), gibberellin (GA), ABA, JA, and SA content (Liu et al., 2016). Nevertheless, these effects may be pleiotropic and may reflect altered general physiological states. As more direct evidence, PRC2 activity in Arabidopsis seed coat is downregulated by fertilization-dependent auxin, and is required for repressing GA production prior to fertilization, mediating the crosstalk between two phytohormonal pathways (Figueiredo et al., 2015, 2016; Figueiredo and Köhler, 2018). PRC2 also represses auxin biosynthesis and signaling genes in the SAM and leaves of Arabidopsis (Lafos et al., 2011). Conversely, in the RAM, the expression of the auxin efflux carrier-encoding *PIN-FORMED* (*PIN*) genes is positively regulated by BRM establishing local auxin maxima and stimulating the expression of the RAM-specifying *PLETHORA* genes *PLT1* and *PLT2* (Yang et al., 2015). BRM also binds to GA-related genes to stimulate GA biosynthesis and signaling (Archacki et al., 2013).

Apart from biosynthesis and signaling, phytohormone-response genes are under direct control of chromatin modifiers. Initially described as involved in auxin homeostasis (Sorin et al., 2005), the ARGONAUTE protein AGO1, guided by small RNAs and associating with SWI/SNF complexes, was recently described to bind genes activated upon JA, auxin, and SA stimuli in Arabidopsis (Liu C. et al., 2018). ABA-responsive genes in Arabidopsis are repressed by histone deacetylation (Perrella et al., 2013) through the action of MULTICOPY SUPRESSOR OF IRA (MSI) 1 recruiting the HISTONE DEACETYLASE (HDA)19 (Alexandre et al., 2009; Mehdi et al., 2016) and also by BRM-mediated chromatin remodeling (Han et al., 2012). Significantly, expression of 80% GA–responsive genes relies on the chromatin remodeler PICKLE (PKL) (Park et al., 2017). Consequently, plants with reduced MSI1, HDA19, or BRM levels are more sensitive to ABA, display ABA-dependent growth defects and higher tolerance to drought, and absence of PKL results in GA-reversible root swelling and embryonic lipid accumulation (Ogas et al., 1997) demonstrating the developmental importance of chromatin modifiers in phytohormone-mediated responses.

## Hormone Signaling and Chromatin Crosstalk can Participate in Plasticity and Robustness

Hormone signaling and chromatin crosstalk can participate in developmental paths by distinct ways: (1) control of cell identity genes in meristems, (2) chromatin-mediated stabilization of gene expression beyond the hormonal initial signal, (3) chromatin-governed integration of separate hormone signaling pathways.

Chromatin-modifying complexes target key phytohormone-regulated genes that specify meristem cell identity and whose ectopic expression can result in cell reprogramming and homeosis (Zuo et al., 2002; Galinha et al., 2007). For example, the SAM-organizing homeobox gene *WUSCHEL* (*WUS*) is regulated by cytokinin signaling, DNA methylation, H3K27me, or chromatin remodeling (Kwon, 2005; Dodsworth, 2009; Cao et al., 2015; Liu H. et al., 2018), and loss of DNA methylation in *WUS* promoter is connected to *in-vitro* shoot initiation induced by cytokinin (Li et al., 2011). Other stem cell niche-defining TFs such as *WOX4, WOX5, PLT1*, or *PLT2* are potential PRC2 targets (Oh et al., 2008; Lafos et al., 2011). Co-expression of these TFs can be triggered by environmental and hormone cues or ectopically induced in PRC2-depleted plants, resulting in cell reprogramming (Chanvivattana et al., 2004; Barrero et al., 2007; Ikeuchi et al., 2015; Mozgová et al., 2017). Increased or dispersed expression of cell identity-defining TFs and change and/or loss of cell identity also occurs in mutants of chromatin modifiers such as the repressive H2A-ubiquitinase complex PRC1 (Xu and Shen, 2008; Bratzel et al., 2010; Chen et al., 2010, 2016), histone deacetylases HDA6 and HAD19 (Tanaka et al., 2008; Pi et al., 2015), PKL (Ogas et al., 1999) or replication-dependent H3/H4 chaperone CHROMATIN ASSEMBLY COMPLEX (CAF)-1 (Kaya et al., 2001). Thus, chromatin structure appears to restrict expression of developmental genes to retain cell identities. Similarly, repression of ABA response by several chromatin modifiers (MSI1, HDA19, BRM) could act to prevent an ectopic stress response in favorable environmental conditions.

Chromatin structure may stabilize gene expression state beyond the duration of the environmental or phytohormone stimulus. An example is the cold-induced establishment of H3K27me3 at *FLC* during vernalization that is stable through mitosis, providing an in-cis memory system of *FLC* repression even after transfer to warmth (Berry et al., 2015; Hepworth and Dean, 2015). Persistent H3K4me2/3, H3/H4ac or local nucleosome depletion are found at genes primed for biotic or abiotic stress responses including priming by phytohormones or their analogs (Jaskiewicz et al., 2011; Lämke and Bäurle, 2017; Laura et al., 2018; Liu H. C. et al., 2018) demonstrating that also “accessible” chromatin structure contributes to mitotic memory (Figure 1).

Chromatin-modifying proteins may also serve as integrators defining the final outcome of interplay of various hormone signaling pathways. Phytohormone-induced change of chromatin structure may rely on multiple different chromatin modifiers as is exemplified by modulators of ABA signaling. A single chromatin modifier can also be implicated in responses to different hormones, as is exemplified by BRM (Sarnowska et al., 2016). Chromatin can thus provide a robust hub integrating different incoming cues while potentiating the persistence of the gene expression patterns through its stability during mitotic cell divisions.

## Meristems are Central Places for Phytohormone Chromatin Crosstalk

The biological significance of the crosstalk in meristems is supported by (1) their central role in postembryonic morphogenesis, plasticity and memory, (2) their particularities for phytohomone signaling and chromatin remodeling, (3) first evidences reported for this crosstalk in SAM.

The meristems represent major sites of stem cell niches in plants (Scheres, 2007; Tucker and Laux, 2007; Aichinger et al., 2012). Apical meristems, together with the secondary meristem, the cambium, have the capacity to maintain and self-renew populations of undifferentiated cells, underlying continuous post-embryonic organ development modulated by environmental conditions (Figure 1; Gaillochet and Lohmann, 2015; Pavlovic and Radotic, 2017; Xiao et al., 2017). The SAM is also the place of epigenetic memory as reported for vernalization and some priming effects (Hepworth and Dean, 2015; Lämke and Bäurle, 2017).

Phytohormone and epigenetic pathways play overlapping/complementary roles in meristem functions and developmental plasticity or robustness, laying the basis for a biologically significant crosstalk. Importantly, meristems have been shown to be the place of epigenetic control for stem cell pluripotency, differentiation, and reprogramming (Cao et al., 2015; Gaillochet and Lohmann, 2015; Pi et al., 2015; Morao et al., 2016; Ojolo et al., 2018) whose epigenetic setup may differ from the surrounding tissues (Yadav et al., 2009; Baubec et al., 2014).

Major evidence for phytohormone-chromatin crosstalk was obtained using Arabidopsis mutants, or applying phytohormones or chemical inhibitors of chromatin modifiers in various developmental processes (Yamamuro et al., 2016; Campos-Rivero et al., 2017; Wong et al., 2017; Guo et al., 2018; Ojolo et al., 2018; Wakeel et al., 2018; Zheng et al., 2018). Only a few reports highlight potential crosstalk directly in the meristems as exemplified by PRC2 repressing particular *PIN* genes (auxin transporters) in the SAM of Arabidopsis *clv3* mutants (Lafos et al., 2011). Recent studies in vernalized sugar beet (Hébrard et al., 2016) and in poplar under drought or cold exposure (Conde et al., 2017; Lafon-Placette et al., 2018; Le Gac et al., 2018) have recently shown that differentially expressed genes under DNA methylation control in SAM correspond to a limited developmental gene network mainly involved in growth and phytohormone pathways such as jasmonate activators and ethylene repressors. Indeed, Le Gac et al. (2018) show that hormone-related epigenome reprogramming in the SAM of poplar hybrids is stable for at least several months after the stress period in winter-dormant SAM providing evidence of an environmental epigenetic memory. Recently, this phenomenon was also described in the SAM of natural populations of black poplar under drought conditions (Sow et al., 2018a). Similarly, support for epigenetic memory of climatic conditions is found in Norway spruce trees grown from somatic embryos produced at different temperatures (Yakovlev et al., 2011, 2016). Considering the absence of post-embryonic organs, the SAM could play a major role in the transmission of the environmentally-established chromatin states during early development.

## Conclusion and Future Perspectives

In conclusion, phytohormone action and chromatin modifiers seem to be tightly interacting but the extent to which they act jointly or independently remains unclear (Ojolo et al., 2018). However, the multi-layered control of local chromatin structure in response to hormonal cues may provide an important hub that integrates the incoming cues, conferring developmental robustness while retaining a sufficient potential for gene transcription change, stabilization and phenotypic plasticity (Lachowiec et al., 2016).

Current knowledge leads to the opinion that this crosstalk in meristems can integrate environmental cues for developmental outcome. Erasure of this signaling may allow continuous adjustment to new environmental conditions. Its maintenance through persistent chromatin states can however stimulate mitotic memory that could prime later organ formation. How the balance between erasure and memory is achieved remains enigmatic (Figure 1).

While the mechanistic events could be more easily deciphered in well-established model annuals such as Arabidopsis, it is important to establish perennial models where the impact of mitotic epigenetic memory is of importance in the context of climate change. In addition to SAM and RAM, cambium, whose activity is crucial for environmentally controlled wood formation, may be an appropriate and highly relevant model (Wang et al., 2016; Oles et al., 2017; Figure 1). Deciphering this crosstalk in the meristems requires improving single-cell methodologies to study the dynamics of chromatin structure in response to complex phytohormone-associated environmental and developmental responses and its memory. Exploiting epigenetic variation and the potential to derive primed plants from meristem regeneration or somatic embryos (Achour et al., 2017; Gallusci et al., 2017; Springer and Schmitz, 2017; Sow et al., 2018b) seems also promising.

## Author Contributions

SM suggested and designed the opinion article. MDS and SM designed the Figure 1. IM and SM finalized and revised the article. All the authors checked and confirmed the final version of the manuscript. All the authors drafted the entire manuscript.

### Conflict of Interest Statement

The authors declare that the research was conducted in the absence of any commercial or financial relationships that could be construed as a potential conflict of interest.

## References

[B1] Achour Z. Archipiano M. Barneche F. Baurens C. Beckert M. Ben C. (2017). Epigenetics in plant breeding, in Article de positionnement du Groupement d'intérêt scientifique Biotechnologies vertes et de l'Alliance nationale de recherche pour l'environnement. Available online at: www.gisbiotechnologiesvertes.com/fr/publications/position-paper-epigenetics-in-plant-breeding (accessed February 13, 2017)

[B2] Aichinger E. Kornet N. Friedrich T. Laux T. (2012). Plant stem cell niches. Ann. Rev. Plant Biol. 63, 615–636. 10.1146/annurev-arplant-042811-105555 22404469

[B3] Alexandre C. Möller-Steinbach Y. Schönrock N. Gruissem W. Hennig L. (2009). Arabidopsis MSI1 is required for negative regulation of the response to drought stress. Mol. Plant 2, 675–687. 10.1093/mp/ssp012 19825648

[B4] Archacki R. Buszewicz D. Sarnowski T. J. Sarnowska E. Rolicka A. T. Tohge T.. (2013). BRAHMA ATPase of the SWI/SNF chromatin remodeling complex acts as a positive regulator of gibberellin-mediated responses in Arabidopsis. PLoS ONE 8:e58588. 10.1371/journal.pone.0058588 23536800 PMC3594165

[B5] Barrero J. M. Gonzalez-Bayon R. del Pozo J. C. Ponce M. R. Micol J. L. (2007). INCURVATA2 encodes the catalytic subunit of DNA polymerase and interacts with genes involved in chromatin-mediated cellular memory in *Arabidopsis thaliana*. Plant Cell 19, 2822–2838. 10.1105/tpc.107.054130 17873092 PMC2048701

[B6] Baubec T. Finke A. Mittelsten Scheid O. Pecinka A. (2014). Meristem-specific expression of epigenetic regulators safeguards transposon silencing in Arabidopsis. EMBO Rep. 15, 446–452. 10.1002/embr.201337915 24562611 PMC3989676

[B7] Berry S. Hartley M. Olsson T. S. G. Dean C. Howard M. (2015). Local chromatin environment of a Polycomb target gene instructs its own epigenetic inheritance. ELife 4:e07205. 10.7554/eLife.07205 25955967 PMC4450441

[B8] Bratzel F. López-Torrejón G. Koch M. Del Pozo J. C. Calonje M. (2010). Keeping cell identity in Arabidopsis requires PRC1 RING-finger homologs that catalyze H2A monoubiquitination. Curr. Biol. 20, 1853–1859. 10.1016/j.cub.2010.09.046 20933424

[B9] Campos-Rivero G. Osorio-Montalvo P. Sánchez-Borges R. Us-Camas R. Duarte-Aké F. De-la-Pe-a C. (2017). Plant hormone signaling in flowering: an epigenetic point of view. J. Plant Physiol. 214, 16–27. 10.1016/j.jplph.2017.03.018 28419906

[B10] Cao X. He Z. Guo L. Liu X. (2015). Epigenetic mechanisms are critical for the regulation of WUSCHEL expression in floral meristems: figure 1. Plant Physiol. 168, 1189–1196. 10.1104/pp.15.00230 25829464 PMC4528737

[B11] Chanvivattana Y. Bishopp A. Schubert D. Stock C. Moon Y.-H. Sung Z. R.. (2004). Interaction of Polycomb-group proteins controlling flowering in Arabidopsis. Development 131, 5263–5276. 10.1242/dev.01400 15456723

[B12] Chen D. Molitor A. Liu C. Shen W.-H. (2010). The Arabidopsis PRC1-like ring-finger proteins are necessary for repression of embryonic traits during vegetative growth. Cell Res. 20, 1332–1344. 10.1038/cr.2010.151 21060339

[B13] Chen D. Molitor A. M. Xu L. Shen W.-H. (2016). Arabidopsis PRC1 core component AtRING1 regulates stem cell-determining carpel development mainly through repression of class I KNOX genes. BMC Biol. 14:112. 10.1186/s12915-016-0336-4 28007029 PMC5178098

[B14] Conde D. Le Gac A.-L. Perales M. Dervinis C. Kirst M. Maury S. (2017). Chilling-responsive DEMETER-LIKE DNA demethylase mediates in poplar bud break: Role of active DNA demethylase in trees' bud break. Plant Cell Environ. 40, 2236–2249. 10.1111/pce.13019 28707409

[B15] Dodsworth S. (2009). A diverse and intricate signalling network regulates stem cell fate in the shoot apical meristem. Dev. Biol. 336, 1–9. 10.1016/j.ydbio.2009.09.031 19782675

[B16] Figueiredo D. D. Batista R. A. Roszak P. J. Hennig L. Köhler C. (2016). Auxin production in the endosperm drives seed coat development in Arabidopsis. ELife 5:e20542. 10.7554/eLife.20542 27848912 PMC5135394

[B17] Figueiredo D. D. Batista R. A. Roszak P. J. Köhler C. (2015). Auxin production couples endosperm development to fertilization. Nat. Plants 1:15184. 10.1038/nplants.2015.184 27251719

[B18] Figueiredo D. D. Köhler C. (2018). Auxin: a molecular trigger of seed development. Genes Dev. 32, 479–490. 10.1101/gad.312546.118 29692356 PMC5959232

[B19] Gaillochet C. Lohmann J. U. (2015). The never-ending story: from pluripotency to plant developmental plasticity. Development 142, 2237–2249. 10.1242/dev.117614 26130755 PMC4510588

[B20] Galinha C. Hofhuis H. Luijten M. Willemsen V. Blilou I. Heidstra R.. (2007). PLETHORA proteins as dose-dependent master regulators of Arabidopsis root development. Nature 449, 1053–1057. 10.1038/nature06206 17960244

[B21] Gallusci P. Dai Z. Génard M. Gauffretau A. Leblanc-Fournier N. Richard-Molard C.. (2017). Epigenetics for plant improvement: current knowledge and modeling avenues. Trends Plant Sci. 22, 610–623. 10.1016/j.tplants.2017.04.009 28587758

[B22] Guo J.-E. Hu Z. Yu X. Li A. Li F. Wang Y.. (2018). A histone deacetylase gene, SlHDA3, acts as a negative regulator of fruit ripening and carotenoid accumulation. Plant Cell Rep. 37, 125–135. 10.1007/s00299-017-2211-3 28932910

[B23] Han S.-K. Sang Y. Rodrigues A. BIOL425 F2010 Wu M.-F. Rodriguez P. L.. (2012). The SWI2/SNF2 chromatin remodeling ATPase BRAHMA represses abscisic acid responses in the absence of the stress stimulus in Arabidopsis. Plant Cell. 24, 4892–4906. 10.1105/tpc.112.105114 23209114 PMC3556964

[B24] Hébrard C. Peterson D. G. Willems G. Delaunay A. Jesson B. Lefèbvre M.. (2016). Epigenomics and bolting tolerance in sugar beet genotypes. J. Exp. Botany 67, 207–225. 10.1093/jxb/erv449 26463996 PMC4682430

[B25] Hepworth J. Dean C. (2015). Flowering Locus C's lessons: conserved chromatin switches underpinning developmental timing and adaptation. Plant Physiol. 168, 1237–1245. 10.1104/pp.15.00496 26149571 PMC4528751

[B26] Ikeuchi M. Iwase A. Rymen B. Harashima H. Shibata M. Ohnuma M.. (2015). PRC2 represses dedifferentiation of mature somatic cells in Arabidopsis. Nat. Plants 1:15089. 10.1038/nplants.2015.89 27250255

[B27] Jaskiewicz M. Conrath U. Peterhänsel C. (2011). Chromatin modification acts as a memory for systemic acquired resistance in the plant stress response. EMBO Rep. 12, 50–55. 10.1038/embor.2010.186 21132017 PMC3024125

[B28] Kaya H. Shibahara K. Taoka K. Iwabuchi M. Stillman B. Araki T. (2001). FASCIATA genes for chromatin assembly factor-1 in Arabidopsis maintain the cellular organization of apical meristems. Cell 104, 131–142. 10.1016/S0092-8674(01)00197-0 11163246

[B29] Kwon C. S. (2005). WUSCHEL is a primary target for transcriptional regulation by SPLAYED in dynamic control of stem cell fate in Arabidopsis. Genes Dev. 19, 992–1003. 10.1101/gad.1276305 15833920 PMC1080137

[B30] Lachowiec J. Queitsch C. Kliebenstein D. J. (2016). Molecular mechanisms governing differential robustness of development and environmental responses in plants. Ann. Botany 117, 795–809. 10.1093/aob/mcv151 26473020 PMC4845800

[B31] Lafon-Placette C. Le Gac A.-L. Chauveau D. Segura V. Delaunay A. Lesage-Descauses M.-C.. (2018). Changes in the epigenome and transcriptome of the poplar shoot apical meristem in response to water availability affect preferentially hormone pathways. J. Exp. Botany 69, 537–551. 10.1093/jxb/erx409 29211860

[B32] Lafos M. Kroll P. Hohenstatt M. L. Thorpe F. L. Clarenz O. Schubert D. (2011). Dynamic regulation of H3K27 trimethylation during Arabidopsis differentiation. PLoS Genet. 7:e1002040. 10.1371/journal.pgen.1002040 21490956 PMC3072373

[B33] Lämke J. Bäurle I. (2017). Epigenetic and chromatin-based mechanisms in environmental stress adaptation and stress memory in plants. Genome Biol. 18:124. 10.1186/s13059-017-1263-6 28655328 PMC5488299

[B34] Latzel V. Zhang Y. Karlsson Moritz K. Fischer M. Bossdorf O. (2012). Epigenetic variation in plant responses to defence hormones. Ann. Botany 110, 1423–1428. 10.1093/aob/mcs088 22543179 PMC3489142

[B35] Laura B. Silvia P. Francesca F. Benedetta S. Carla C. (2018). Epigenetic control of defense genes following MeJA-induced priming in rice (*O. sativa*). J. Plant Physiol. 228, 166–177. 10.1016/j.jplph.2018.06.007 29936261

[B36] Le Gac A.-L. Lafon-Placette C. Chauveau D. Segura V. Delaunay A. Fichot R.. (2018). Winter-dormant shoot apical meristem in poplar trees shows environmental epigenetic memory. J. Exp. Botany 69, 4821–4837. 10.1093/jxb/ery271 30107545 PMC6137975

[B37] Li Q.-F. Lu J. Yu J.-W. Zhang C.-Q. He J.-X. Liu Q.-Q. (2018). The brassinosteroid-regulated transcription factors BZR1/BES1 function as a coordinator in multisignal-regulated plant growth. Biochim. Biophys. Acta Gene Regul. Mech. 1861, 561–571. 10.1016/j.bbagrm.2018.04.003 29673687

[B38] Li W. Liu H. Cheng Z. J. Su Y. H. Han H. N. Zhang Y.. (2011). DNA methylation and histone modifications regulate de novo shoot regeneration in Arabidopsis by modulating WUSCHEL expression and auxin signaling. PLoS Genet. 7:e1002243. 10.1371/journal.pgen.1002243 21876682 PMC3158056

[B39] Liu C. Xin Y. Xu L. Cai Z. Xue Y. Liu Y.. (2018). Arabidopsis ARGONAUTE 1 binds chromatin to promote gene transcription in response to hormones and stresses. Dev. Cell 44, 348–361.e7. 10.1016/j.devcel.2017.12.002 29290588

[B40] Liu H. Zhang H. Dong Y. X. Hao Y. J. Zhang X. S. (2018). DNA METHYLTRANSFERASE1 -mediated shoot regeneration is regulated by cytokinin-induced cell cycle in Arabidopsis. N. Phytol. 217, 219–232. 10.1111/nph.14814 28960381

[B41] Liu H. C. Lämke J. Lin S. Hung M.-J. Liu K.-M. Charng Y. Bäurle I. (2018). Distinct heat shock factors and chromatin modifications mediate the organ-autonomous transcriptional memory of heat stress. Plant J. 95, 401–413. 10.1111/tpj.13958 29752744

[B42] Liu X. Wei X. Sheng Z. Jiao G. Tang S. Luo J.. (2016). Polycomb protein OsFIE2 affects plant height and grain yield in rice. PLoS ONE 11:e0164748. 10.1371/journal.pone.0164748 27764161 PMC5072591

[B43] Mehdi S. Derkacheva M. Ramström M. Kralemann L. Bergquist J. Hennig L. (2016). The WD40 domain protein MSI1 functions in a HDAC complex to fine-tune ABA signaling. Plant Cell 28, 42–54. 10.1105/tpc.15.00763 26704384 PMC4746680

[B44] Morao A. K. Bouyer D. Roudier F. (2016). Emerging concepts in chromatin-level regulation of plant cell differentiation: timing, counting, sensing and maintaining. Curr. Opin. Plant Biol. 34, 27–34. 10.1016/j.pbi.2016.07.010 27522467

[B45] Mozgová I. Mu-oz-Viana R. Hennig L. (2017). PRC2 represses hormone-induced somatic embryogenesis in vegetative tissue of *Arabidopsis thaliana*. PLoS Genet. 13:e1006562. 10.1371/journal.pgen.1006562 28095419 PMC5283764

[B46] Ogas J. Cheng J.-C. Sung Z.R. Somerville C. (1997). Cellular differentiation regulated by gibberellin in the *Arabidopsis thaliana* pickle mutant. Sciences 277, 91–94. 10.1126/science.277.5322.91 9204906

[B47] Ogas J. Kaufmann S. Henderson J. Somerville C. (1999). PICKLE is a CHD3 chromatin-remodeling factor that regulates the transition from embryonic to vegetative development in Arabidopsis. Proc. Natl. Acad. Sci. U.S.A. 96, 13839–13844. 10.1073/pnas.96.24.13839 10570159 PMC24151

[B48] Oh S. Park S. van Nocker S. (2008). Genic and global functions for Paf1C in chromatin modification and gene expression in Arabidopsis. PLoS Genet. 4:e1000077. 10.1371/journal.pgen.1000077 18725930 PMC2515192

[B49] Ojolo S. P. Cao S. Priyadarshani S. V. G. N. Li W. Yan M. Aslam M.. (2018). Regulation of plant growth and development: a review from a chromatin remodeling perspective. Front. Plant Sci. 9:1232. 10.3389/fpls.2018.01232 30186301 PMC6113404

[B50] Oles V. Panchenko A. Smertenko A. (2017). Modeling hormonal control of cambium proliferation. PLoS ONE 12:e0171927. 10.1371/journal.pone.0171927 28187161 PMC5302410

[B51] Park J. Oh D.-H. Dassanayake M. Nguyen K. T. Ogas J. Choi G.. (2017). Gibberellin signaling requires chromatin remodeler PICKLE to Promote vegetative growth and phase transitions. Plant Physiol. 173, 1463–1474. 10.1104/pp.16.01471 28057895 PMC5291033

[B52] Pavlovic M. Radotic K. (2017). Animal and Plant Stem Cells, Vol. 234 Cham: Springer International Publishing XVII.

[B53] Peirats-Llobet M. Han S.-K. Gonzalez-Guzman M. Jeong C. W. Rodriguez L. Belda-Palazon B.. (2016). A direct link between abscisic acid sensing and the chromatin-remodeling ATPase BRAHMA via core ABA signaling pathway components. Mol. Plant 9, 136–147. 10.1016/j.molp.2015.10.003 26499068

[B54] Perrella G. Lopez-Vernaza M. A. Carr C. Sani E. Gossele V. Verduyn C.. (2013). Histone deacetylase complex1 expression level titrates plant growth and abscisic acid sensitivity in Arabidopsis. Plant Cell 25, 3491–3505. 10.1105/tpc.113.114835 24058159 PMC3809545

[B55] Pi L. Aichinger E. van der Graaff E. Llavata-Peris C. I. Weijers D. Hennig L.. (2015). Organizer-derived WOX5 signal maintains root columella stem cells through chromatin-mediated repression of CDF4 expression. Dev. Cell 33, 576–588. 10.1016/j.devcel.2015.04.024 26028217

[B56] Sarnowska E. Gratkowska D. M. Sacharowski S. P. Cwiek P. Tohge T. Fernie A. R.. (2016). The role of SWI/SNF chromatin remodeling complexes in hormone crosstalk. Trends Plant Sci. 21, 594–608. 10.1016/j.tplants.2016.01.017 26920655

[B57] Scheres B. (2007). Stem-cell niches: nursery rhymes across kingdoms. Nat. Rev. Mol. Cell Biol. 8, 345–354. 10.1038/nrm2164 17450175

[B58] Sorin C. Bussell J. D. Camus I. Ljung K. Kowalczyk M. Geiss G.. (2005). Auxin and light control of adventitious rooting in Arabidopsis require ARGONAUTE1. Plant Cell 17, 1343–1359. 10.1105/tpc.105.031625 15829601 PMC1091759

[B59] Sow M. D. Allona I. Ambroise C. Conde D. Fichot R. Gribkova S. (2018b). Epigenetics in forest trees, in Advances in Botanical Research. Plant Epigenetics Coming of Age for Breeding Applications, Vol. 88 éds Gallusci P. Bucher E. Mirouze M. 387–453. Academic Press, Elsevier 10.1016/bs.abr.2018.09.003

[B60] Sow M. D. Segura V. Chamaillard S. Jorge V. Delaunay A. Lafon-Placette C. (2018a). Narrow-sense heritability and PST estimates of DNA methylation in three *Populus nigra* L. populations under contrasting water availability. Tree Genet. Genomes 14:78 10.1007/s11295-018-1293-6

[B61] Springer N. M. Schmitz R. J. (2017). Exploiting induced and natural epigenetic variation for crop improvement. Nat. Rev. Genet. 18, 563–575. 10.1038/nrg.2017.45 28669983

[B62] Tanaka M. Kikuchi A. Kamada H. (2008). The Arabidopsis Histone Deacetylases HDA6 and HDA19 contribute to the repression of embryonic properties after germination. Plant Physiol. 146, 149–161. 10.1104/pp.107.111674 18024558 PMC2230551

[B63] Tucker M. R. Laux T. (2007). Connecting the paths in plant stem cell regulation. Trends Cell Biol. 17, 403–410. 10.1016/j.tcb.2007.06.002 17766120

[B64] Wakeel A. Ali I. Khan A. R. Wu M. Upreti S. Liu D.. (2018). Involvement of histone acetylation and deacetylation in regulating auxin responses and associated phenotypic changes in plants. Plant Cell Rep. 37, 51–59. 10.1007/s00299-017-2205-1 28948334

[B65] Wang Q. Ci D. Li T. Li P. Song Y. Chen J.. (2016). The role of DNA methylation in xylogenesis in different tissues of poplar. Front. Plant Sci. 7:1003. 10.3389/fpls.2016.01003 27462332 PMC4941658

[B66] Wong M. M. Chong G. L. Verslues P. E. (2017). Epigenetics and RNA processing: connections to drought, salt, and ABA?, in Plant Stress Tolerance: Methods and Protocols, ed Sunkar R. (New York, NY: Springer New York), 3–21. 10.1007/978-1-4939-7136-7_1 28735388

[B67] Xiao J. Jin R. Wagner D. (2017). Developmental transitions: integrating environmental cues with hormonal signaling in the chromatin landscape in plants. Genome Biol. 18:88. 10.1186/s13059-017-1228-9 28490341 PMC5425979

[B68] Xu L. Shen W.-H. (2008). Polycomb silencing of KNOX genes confines shoot stem cell niches in Arabidopsis. Curr. Biol. 18, 1966–1971. 10.1016/j.cub.2008.11.019 19097900

[B69] Yadav R. K. Girke T. Pasala S. Xie M. Reddy G. V. (2009). Gene expression map of the Arabidopsis shoot apical meristem stem cell niche. Proc. Natl. Acad. Sci. U.S.A. 106, 4941–4946. 10.1073/pnas.0900843106 19258454 PMC2660727

[B70] Yakovlev I. A. Asante D. K. A. Fossdal C. G. Junttila O. Johnsen Ø. (2011). Differential gene expression related to an epigenetic memory affecting climatic adaptation in Norway spruce. Plant Sci. 180, 132–139. 10.1016/j.plantsci.2010.07.004 21421355

[B71] Yakovlev I. A. Carneros E. Lee Y. Olsen J. E. Fossdal C. G. (2016). Transcriptional profiling of epigenetic regulators in somatic embryos during temperature induced formation of an epigenetic memory in Norway spruce. Planta 243, 1237–1249. 10.1007/s00425-016-2484-8 26895338

[B72] Yamamuro C. Zhu J.-K. Yang Z. (2016). Epigenetic modifications and plant hormone action. Mol. Plant 9, 57–70. 10.1016/j.molp.2015.10.008 26520015 PMC5575749

[B73] Yang S. Li C. Zhao L. Gao S. Lu J. Zhao M.. (2015). The Arabidopsis SWI2/SNF2 chromatin remodeling ATPase BRAHMA targets directly to PINs and is required for root stem cell niche maintenance. Plant Cell 27, 1670–1680. 10.1105/tpc.15.00091 25991732 PMC4498203

[B74] Yu X. Li L. Li L. Guo M. Chory J. Yin Y. (2008). Modulation of brassinosteroid-regulated gene expression by jumonji domain-containing proteins ELF6 and REF6 in Arabidopsis. Proc. Natl. Acad. Sci. U.S.A. 105, 7618–7623. 10.1073/pnas.0802254105 18467490 PMC2396691

[B75] Zheng X. Hou H. Zhang H. Yue M. Hu Y. Li L. (2018). Histone acetylation is involved in GA-mediated 45S rDNA decondensation in maize aleurone layers. Plant Cell Rep. 37, 115–123. 10.1007/s00299-017-2207-z 28939922

[B76] Zuo J. Niu Q.-W. Frugis G. Chua N.-H. (2002). The WUSCHEL gene promotes vegetative-to-embryonic transition in Arabidopsis. Plant J. 30, 349–359. 10.1046/j.1365-313X.2002.01289.x 12000682

